# Rethinking estrogen receptor β in EAOC: a synergistic modulator in a genomically informed landscape

**DOI:** 10.1186/s13048-026-01990-6

**Published:** 2026-01-27

**Authors:** Li Luo, Weiwei Dai, Na Cao, Mingzhu Ye

**Affiliations:** Department of Obstetrics and Gynecology, Zhongshan Hospital of Xiamen University, School of Medicine, Xiamen University, Xiamen, China

**Keywords:** Endometriosis-associated ovarian cancer (EAOC), Estrogen receptor β (ERβ), BDNF/TrkB signaling pathway, PI3K/AKT pathway, ARID1A, Driver mutations, Molecular subtyping

## Abstract

Endometriosis-associated ovarian cancer (EAOC), encompassing subtypes like ovarian clear cell (OCCC) and endometrioid (OEC) carcinoma, represents a distinct Type I malignancy arising from endometriotic lesions. These tumors are characterized by a specific molecular landscape, including high-frequency driver mutations in genes such as ARID1A, PIK3CA, and PTEN. Within this setting, the role of estrogen receptor β (ERβ), whose expression is progressively upregulated during malignant transformation, requires a nuanced re-evaluation. This review repositions ERβ not as a primary oncogenic driver, but as a critical, spatiotemporal modulator. Its principal function appears to be potentiating pro-survival signaling, such as the PI3K/AKT pathway, within a cellular environment already primed by constitutive genetic alterations. Furthermore, ERβ appears to couple apoptosis resistance with microenvironmental remodeling and metastatic programming. We further dissect the role of the downstream ERβ–brain-derived neurotrophic factor (BDNF)/Tropomyosin receptor kinase B (TrkB) signaling axis, proposing it as a key cooperative network that provides parallel and compensatory survival signals. The central thesis is that the significance of this axis is profoundly context-dependent, and its roles should be interpreted alongside the tumor’s underlying genomic status. Finally, we outline translational prospects, arguing that targeting this pathway will require precision medicine strategies, including composite biomarkers and rational combination therapies. These strategies should be tailored to the specific molecular subtype of each patient’s tumor.

## Introduction

Endometriosis is a common, hormone-dependent gynecologic disease characterized by the presence of endometrial-like tissue outside the uterine cavity [1]. While histologically benign, it exhibits malignancy-like behaviors and is recognized as a precursor for specific subtypes of epithelial ovarian cancer (EOC) [2, 3]. The classification of EOC into two major clinicopathological and molecular frameworks, Type I and Type II, is essential for understanding this malignant transformation [4, 5]. Type I tumors, which include ovarian clear cell carcinoma (OCCC) and endometrioid carcinoma (OEC), are typically low-grade, genetically stable, and progress stepwise from identifiable precursor lesions [6]. They frequently harbor specific driver mutations in genes such as ARID1A, PIK3CA, PTEN, and KRAS [7]. In contrast, Type II tumors, dominated by high-grade serous carcinoma, are highly aggressive, genetically unstable, and almost universally defined by mutations in TP53 [5]. This review focuses on Type I EAOCs, which present distinct therapeutic challenges due to their relative chemoresistance compared to Type II tumors [8]. It is within the Type I framework that the etiological link between endometriosis and ovarian cancer becomes clear. Large-scale cohort studies report that the standardized incidence ratios (SIR) for developing OCCC and OEC in patients with endometriosis are significantly elevated, at 3.05 (95% CI, 2.43–3.83) and 2.04 (95% CI, 1.67–2.48), respectively [1]. These estimates vary by population, follow-up, and ascertainment; we therefore treat them as directional evidence of risk enrichment rather than precise prognostic coefficients [2]. These EAOCs often exhibit direct histological continuity with endometriotic lesions, underscoring their origin from this precursor [9]. The anatomical context is also important: endometrioid carcinoma is categorized as a Type I EOC when arising in the ovary, a common site for EAOC [10]. This is distinct from endometrial carcinoma originating in the uterus, which follows a different pathogenic pathway [11].

Against this backdrop of Type I oncogenesis, the role of the estrogen signaling axis, particularly estrogen receptor β (ERβ), has garnered significant attention [12]. In stark contrast to the progressive downregulation of estrogen receptor α (ERα), ERβ expression appears to be activated in early endometriotic lesions and is continuously upregulated in atypical lesions and EAOC [13, 14]. This dynamic expression pattern suggests ERβ may serve as a signaling hub that facilitates malignant progression [15]. This review will synthesize the current understanding of Erβ’s role in EAOC, repositioning it not as a primary driver, but as a critical modulator that synergizes with the established genetic landscape of Type I tumors. We will specifically examine the mechanisms through which ERβ and the BDNF/TrkB axis may amplify oncogenic signals and propose a framework for translational investigation based on genomic stratification.

## Spatiotemporal expression and functional mechanisms of ERβ

### Expression dynamics and contextual caveats

**Fig. 1:**
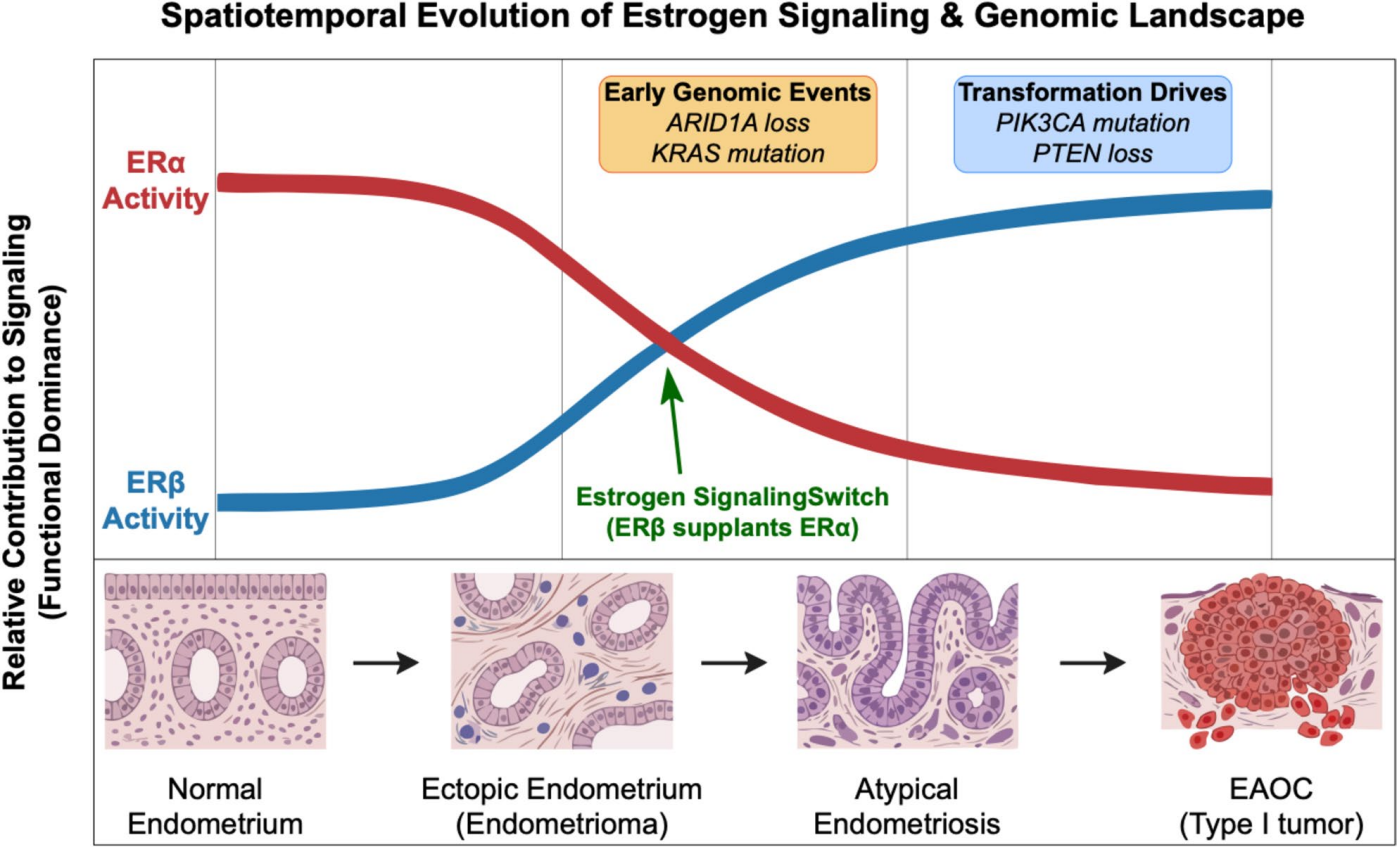
The spatiotemporal evolution of estrogen signaling and genomic alterations in the progression from endometriosis to EAOC. The transition from benign endometriosis to Type I EAOC is characterized by distinct molecular shifts occurring against a backdrop of accumulating driver mutations. As illustrated, while ERα activity (red line) is progressively downregulated, ERβ activity (blue line) is activated early in endometriotic lesions and further upregulated during the transition to atypical endometriosis and carcinoma, creating an “estrogen receptor switch”. Concurrently, ARID1A loss and KRAS mutations often emerge as early events in ectopic lesions, while PIK3CA activating mutations and PTEN loss consolidate the malignant transformation, creating a “genomically primed” environment for ERβ-mediated signaling. Tissue morphology schematics at the bottom illustrate the histological progression from normal endometrium to invasive carcinoma

The pathogenesis of EAOC is intrinsically linked to a dynamic imbalance in the estrogen signaling axis [16]. As a critical transcription factor, the expression dynamics of ERβ exhibit a significant spatiotemporal pattern [17]. A systematic transcriptome analysis by Andersen et al. [15] reported a pronounced stepwise increase in ERβ expression across the continuum from normal endometrium, endometriotic lesions, and atypical hyperplastic lesions, to EAOC tissues (Fig. 1). This trajectory indicates that ERβ transcriptional activation may be an early and sustained event [18]. From a molecular perspective, Trukhacheva et al. [19] demonstrated that highly expressed ERβ can reduce basal ERα expression in endometriotic stromal cells, suggesting the ERβ-regulated network may gradually supplant the ERα-mediated pathway. This must also be interpreted within a spatial context, as ERβ activation appears particularly pronounced within ovarian endometriomas, suggesting the unique ovarian microenvironment may provide specific cues, such as iron-driven oxidative stress and an IL-6/STAT3 inflammatory signaling axis in endometriomas [20, 21].

It is crucial, however, to acknowledge several contextual caveats. The literature contains conflicting reports on Erβ’s role [22]. This heterogeneity likely stems from the existence of multiple ERβ splice variants (ERβ1–5) with differing functions, as well as the varying specificity of antibodies used in immunohistochemistry (IHC) studies [16]. ERβ1 is generally recognized as the principal transcriptionally active isoform, whereas ERβ2 and ERβ5 have been reported to act as dominant-negative regulators [23, 24]. Therefore, while the predominant evidence in the EAOC context points towards a pro-tumorigenic role, this interpretation requires careful calibration. Future studies should prioritize ERβ1-resolved assays with validated antibody clones and employ spatial transcriptomics to capture endometrioma-adjacent heterogeneity [8]. For clarity, the structural features and functional roles of the principal ERβ isoforms are summarized in Table 1.

**Table 1:** ERβ isoforms and related signaling partners (TrkB, ERα) in the EAOC context

Isoform	Structural/conserved-domain summary	Core functional tendency	Canonical pathways/interactions
ERβ1	DBD+/LBD + intact; AF-1/AF-2 retained; classical ligand-binding pocket and dimer interface preserved [16]	Functional; amplifies pro-survival and anti-apoptotic signals in pre-activated contexts [61]	ERE-dependent transcription [37]; cooperates with AP-1/Sp1 [44]; couples with PI3K/AKT and MAPK cascades [26]
ERβ2 (ERβcx)	C-terminal alternative splicing alters LBD/AF-2; DBD retained; tends to heterodimerize [16]	Dominant-negative/antagonistic towards ERβ1 [24]	Shifts dimer composition and weakens classical ERE-driven transcription [24]
ERβ5	Partial loss/alteration of LBD; helix-12 abnormal; AF-2 limited; DBD retained [16]	Weak activation or non-classical/antagonistic modulation [24]	Bias towards non-classical regulation; modulates proliferation/migration-related networks [23]
TrkB (NTRK2)	Membrane RTK with extracellular Ig-like domains and a transmembrane segment; full-length kinase-active vs. truncated isoforms [53]	BDNF-axis co-amplifier/bypass activator [39]	Activates PI3K/AKT and MAPK/ERK [54]; provides a bypass amplification loop [55]
ERα (reference)	DBD+/LBD + intact; AF-1/AF-2 retained [45]	Contextual reference in EAOC	Often down-regulated in EAOC [15]; indicates pathway re-programming [19]

*Abbreviations*: *AF-1/2* Activation Function 1/2, *AKT* Protein Kinase B, *AP-1* Activator Protein 1, *BDNF* Brain-Derived Neurotrophic Factor, *cx* C-terminal exchange, *DBD* DNA-Binding Domain, *EAOC* Endometriosis-Associated Ovarian Cancer, *ER* Estrogen Receptor, *ERE* Estrogen Response Element, *ERK* Extracellular Signal-Regulated Kinase, *Ig*-*like* Immunoglobulin-like, *LBD* Ligand-Binding Domain, *MAPK* Mitogen-Activated Protein Kinase, *NTRK2* Neurotrophic Receptor Tyrosine Kinase 2, *PI3K* Phosphoinositide 3-Kinase, *RTK* Receptor Tyrosine Kinase, *Sp1* Specificity Protein 1, *TrkB* Tropomyosin Receptor Kinase B

### Amplifying pro-survival signaling in a genomically primed context

The functional contribution of ERβ to EAOC pathogenesis is best understood not as a primary oncogenic driver, but as a multifaceted modulator that potentiates pro-tumorigenic phenotypes. The PI3K/AKT pathway is constitutively activated in a large fraction of EAOCs, often initiated by intrinsic structural alterations such as PTEN loss or PIK3CA activating mutations [25]. Within this genetically primed setting, ERβ may function as a potent secondary amplifier. Findings from other contexts, such as those by Hu et al. [26] in non-small cell lung cancer, suggest a model where ERβ may transcriptionally repress PTEN expression. This is consistent with enhancing pro-survival signaling, relieving negative regulation of the PI3K/AKT pathway, upregulating BCL-2 and CCND1, and leading to marked reductions in apoptosis in vitro [27, 28]. While translatable mechanisms are plausible, EAOC-specific validation remains essential. This ERβ-mediated repression of PTEN could synergize with high-frequency genetic events like PIK3CA activating mutations, creating a positive feedback loop [29]. For instance, it can be hypothesized that in a PIK3CA-mutant EAOC cell, a concurrent ERβ-mediated repression of the remaining wild-type PTEN allele would ensure maximal pathway activation. This can be tested using CRISPR-mediated ERβ perturbation in genotype-matched EAOC organoids (PIK3CA-mutant, PTEN-loss, and wild-type controls) with prespecified readouts (p-AKT, BCL-2, CCND1, and Annexin V/cleaved caspase) [30].

### Remodeling the tumor microenvironment and programming a metastatic phenotype

Beyond intracellular signaling, ERβ appears to influence the tumor microenvironment and cellular plasticity [31]. Supportive evidence from an immortalized human endometriotic epithelial cell (iHEEC) model by Han et al. [28] revealed that ERβ overexpression confers resistance to TNF-α-induced apoptosis by blocking the caspase-8/caspase-3 cascade. This link is notable because it connects apoptosis resistance with pro-tumor inflammation and epithelial plasticity. This mechanism is coupled with the upregulation of pro-inflammatory cytokines (e.g., IL-1β) and epithelial–mesenchymal transition (EMT)-driving transcription factors like Slug and Snail. This is consistent with reports from other models, such as Di Zazzo et al. [32] in prostate cancer, where ERβ was shown to drive EMT by modulating E-cadherin, N-cadherin, and Vimentin. Together with the iron-rich, inflammatory endometrioma niche, these programs plausibly reinforce one another to promote invasion [33]. Crucially, this iron-accumulated microenvironment imposes severe oxidative stress, creating a selective pressure that typically triggers ferroptosis [34]. We postulate that ERβ signaling confers ferroptosis resistance by transcriptionally upregulating antioxidant defenses such as glutathione peroxidase 4 (GPX4), a mechanism recently validated in other epithelial models [35]. This adaptation enables EAOC cells to survive metabolic stress while simultaneously driving an invasive phenotype. Within this unique ecological niche, ERβ’s function may be particularly amplified [27]. Time-resolved phosphoproteomics, together with cytokine neutralization/rescue (e.g., IL-1β or IL-6), would help distinguish ERβ-centered programs from stress-induced EMT [36].

## The ERβ–BDNF/TrkB axis: a cooperative signaling network

As the spatiotemporal upregulation of ERβ in EAOC becomes better understood (as detailed in Sect. 2), research focus has expanded to identifying its critical downstream effector networks. Given the established role of ERβ as a transcriptional regulator of neurotrophins in the central nervous system, growing evidence suggests that this signaling module is functionally conserved and repurposed to drive malignancy within the ovarian tumor microenvironment [37]. Within this context, the axis formed by brain-derived neurotrophic factor (BDNF) and its high-affinity receptor, TrkB (NTRK2), has emerged as a key mediator [38, 39]. Based on accumulating data, we propose that this axis functions not merely as a linear conduit of ERβ activity, but as a cooperative node that amplifies survival signals in a context-dependent manner [40, 41]. To substantiate this model, we first examine the mechanistic evidence linking ERβ to BDNF regulation before discussing its functional consequences in EAOC.

### Potential mechanisms of ERβ-mediated BDNF regulation

The hypothesis that ERβ drives this signaling axis hinges on its ability to directly or indirectly regulate BDNF expression [42]. Evidence, primarily from neuronal and other cancer models, suggests this occurs through a multi-layered regulatory network [43]. At the transcriptional level, this may involve direct binding; Yan et al. [37] used CUT&Tag in primary mouse neurons and confirmed that ERβ can selectively bind to a canonical ERE within the BDNF promoter. A nonclassical “tethering” model with the AP-1 complex is also plausible, supported by findings from Indukuri et al. [44] in colorectal cancer cells, where ERβ-bound regions are highly enriched for the AP-1 motif.

Epigenetic regulation represents another critical layer [45]. Studies in rodent models have demonstrated that E2 can induce chromatin remodeling at the BDNF promoter via its receptors [46]. This includes a 3.1-fold increase in H3K4 trimethylation and a 2.4-fold increase in H3K9 acetylation, accompanied by co-recruitment of CBP/p300 (4.2-fold) and MLL3 (3.7-fold). Finally, control may extend to the post-transcriptional level [23]. Given that ERβ can modulate miRNA profiles, it is plausible that ERβ could relieve repression of BDNF mRNA (e.g., by miR-206) by downregulating such miRNAs, thereby stabilizing the BDNF transcript [47]. While these mechanisms provide a strong biological rationale, they have not yet been directly validated within the EAOC cellular context. CRISPRi/a targeting promoter-proximal ERE/AP-1 motifs would provide locus-specific causal evidence. Furthermore, it remains a critical, testable hypothesis whether ARID1A loss, common in EAOC, alters BDNF promoter accessibility, thereby enhancing ERβ or AP-1 “tethering”. This could be investigated using CUT&Tag and ATAC-seq in genotype-matched models [48].

### Context-dependent functional role in EAOC

With the regulatory link established, the functional interplay of this axis with its downstream effectors must be interpreted through the lens of EAOC’s molecular heterogeneity. The binding of BDNF to TrkB triggers a pleiotropic signaling cascade [39]. Evidence from non-EAOC models suggests this axis engages PI3K/AKT, MAPK/ERK, and JAK/STAT pathways [49]. For example, Yuan et al. [50] associated high BDNF/TrkB expression with an intensified invasive phenotype (OR = 3.2; 95% CI, 1.8–5.7) and lymph node metastasis (HR = 2.4; *p* = 0.003) in cervical cancer. Kang et al. [51] confirmed BDNF supports the malignant phenotype by reducing anoikis (anoikis resistance rate: 68% vs. 23%) in a fallopian tube epithelium (FTE) cell model.

**Fig. 2:**
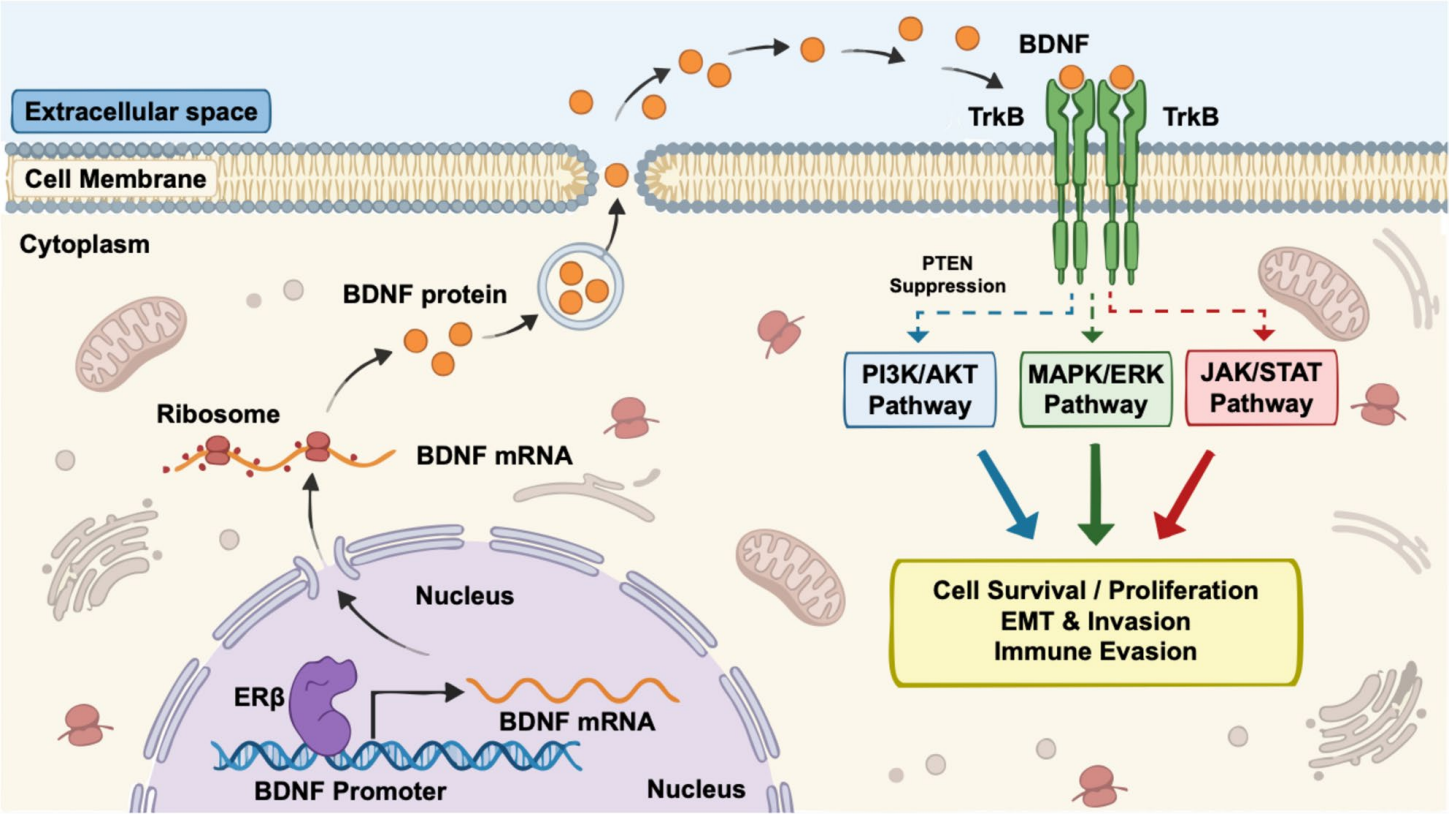
Mechanistic model of the ERβ–BDNF/TrkB cooperative signaling network in EAOC cells. This schematic illustrates the autocrine signaling loop driven by ERβ within the ovarian tumor microenvironment. Mechanistically, nuclear ERβ upregulates BDNF transcription through direct ERE binding or AP-1 tethering, leading to the translation and secretion of BDNF protein via exocytosis. Subsequently, extracellular BDNF acts as an autocrine ligand, binding to and activating the membrane-bound TrkB receptor through dimerization and phosphorylation. This triggers pleiotropic downstream cascades, including PI3K/AKT, MAPK/ERK, and JAK/STAT, which are further modulated by the tumor’s intrinsic genomic context (e.g., PTEN loss). Collectively, the network converges to drive aggressive malignant phenotypes, including cell survival, EMT, invasion, and immune evasion

Within EAOC, the pathological role of this pathway is increasingly apparent [52]. Investigating OCCC, Goto et al. [53] reported that high expression of the TrkB-TK (full-length) isoform was associated with late-stage disease (85.7% vs. 50.0%), deep stromal invasion (OR = 3.4; 95% CI, 1.6–7.1), platinum resistance (a 4.3-fold increase in IC50 for cisplatin), and shorter progression-free survival (HR = 2.3; *p* = 0.008). Given that a large subset of these Type I tumors harbors intrinsic mutations in PIK3CA or loss of PTEN [49], we propose a nuanced, dichotomous model (Fig. 2). On the one hand, BDNF/TrkB functions as a synergistic amplifier in PIK3CA-mutant or PTEN-loss tumors by engaging MAPK/ERK and JAK/STAT signaling [54], yielding dual phospho-signatures (p-AKT with p-ERK/JAK–STAT) and exhibiting combination-therapy synergy [55]; while, on the other hand, in PIK3CA/PTEN wild-type tumors, ERβ-driven BDNF/TrkB operates as an upstream initiator of PI3K/AKT, marked by PI3K-proximal activation and preferential sensitivity to upstream blockade. This framework allows for the formulation of specific, testable hypotheses regarding therapeutic stratification.

## Clinical significance and translational prospects: a context-dependent view

The clinical utility of the ERβ–BDNF/TrkB signaling axis cannot be evaluated in isolation [56]. Its relevance is intrinsically tied to the molecular heterogeneity of the disease [57].

### Re-evaluating biomarker potential in a heterogeneous disease

While the progressive upregulation of ERβ and BDNF suggests biomarker potential [18], a simplistic “dual-high” expression phenotype is likely insufficient as a predictor in Type I tumors. A more promising avenue is the development of a composite biomarker panel. A minimal prototype might include: (1) ERβ/TrkB IHC scores to establish target presence, (2) p-AKT/p-ERK IHC as functional pathway activity readouts, and (3) a simplified transcriptomic signature (e.g., BDNF, NTRK2, EGR1/AP-1 targets). Cutoffs should be derived in a discovery cohort and locked before validation to avoid optimism bias. Validation requires a stepwise approach: from retrospective cohorts to prospective biobanks, integrated into genotype-stratified multivariate models that account for PIK3CA and ARID1A status [36].

### Towards a genomically informed therapeutic strategy

This context-dependent view has profound implications for therapy. Since FDA-approved Trk inhibitors (e.g., Larotrectinib) are indicated for NTRK fusions, which are rare in EAOC, their use here represents an off-label application targeting non-fusion, ligand-dependent bypass signaling [55, 58]. A genomically informed approach is essential and would begin with stratifying patients by PIK3CA, PTEN, and ARID1A status. In PIK3CA/PTEN wild-type disease, we hypothesize that ERβ or TrkB inhibition as monotherapy could be effective where this pathway acts as a critical driver [59]. Whereas, in PIK3CA-mutant or PTEN-loss tumors, rational combinations, i.e., pairing a PI3K inhibitor with a TrkB inhibitor, should be prioritized to block both the primary driver pathway and a compensatory bypass route [60]. This rationale is supported by preclinical data from Goto et al. [53], where TrkB inhibition re-sensitized OCCC cells to cisplatin. Accordingly, prospective trials should deploy the composite panel outlined above to identify patients most likely to benefit.

## Conclusion and future perspectives

This review reframes ERβ as a spatiotemporal amplifier rather than a primary oncogenic driver in EAOC. Within genomically primed backgrounds—most notably tumors harboring PIK3CA and PTEN lesions or ARID1A loss—ERβ appears to potentiate PI3K/AKT–MAPK signaling, coupling apoptosis resistance to epithelial–mesenchymal plasticity, and promoting microenvironmental remodeling with pro-metastatic features. The ERβ–BDNF/TrkB axis offers a coherent conduit linking endocrine cues to neurotrophin pathways, providing a mechanistic rationale for the context-dependent effects observed across ovarian clear cell and endometrioid histotypes. Conceptually, this model unifies disparate findings by positioning ERβ as a permissive modulator whose phenotypic output is strictly gated by genotype, cell state, and the stromal–immune context.

However, bridging the gap between this biological framework and clinical application requires addressing significant translational hurdles. Several caveats, including isoform heterogeneity and the variability of antibody specificity in historical literature, temper immediate adoption. Therefore, future efforts must move beyond simplistic expression metrics. Translational success will depend on establishing standardized, benchmarked assays for ERβ and TrkB, supported by orthogonal phospho-proteomic verification to ensure inter-laboratory comparability.

To convert plausibility into proof, a rigorous research agenda is essential. In parallel with assay standardization, mechanistic dissection should be pursued in genotype-matched systems, such as CRISPR-engineered organoids stratified by ARID1A and PIK3CA/PTEN status. These models are critical for quantifying exactly how the ERβ–BDNF/TrkB axis influences cellular plasticity and drug sensitivity. Building on these insights, preclinical studies must prioritize rational combinations that pair endocrine modulation or TrkB interference with pathway-directed agents, using pharmacodynamic endpoints to validate the proposed mechanism. Ultimately, these data should inform biomarker-enriched, early-phase clinical trials that co-stratify patients by pathway activity and genomic alterations, thereby accelerating the development of precision therapies for women with EAOC.

## Data Availability

No datasets were generated or analysed during the current study.

## Abbreviations

EOC: Epithelial ovarian cancer
OCCC: Ovarian clear cell carcinoma
OEC: Endometrioid carcinoma
ARID1A: AT-rich interactive domain-containing protein 1A
PIK3CA: Phosphatidylinositol-4,5-bisphosphate 3-kinase catalytic subunit alpha
PTEN: Phosphatase and tensin homolog
KRAS: KRAS proto-oncogene
TP53: Tumor protein p53
EAOC: Endometriosis-associated ovarian cancer
SIR: Standardized incidence ratio
CI: Confidence interval
ERβ: Estrogen receptor beta
ERα: Estrogen receptor alpha
BDNF: Brain-derived neurotrophic factor
TrkB: Tropomyosin receptor kinase B (NTRK2)
IHC: Immunohistochemistry
ERE: Estrogen response element
RTK: Receptor tyrosine kinase
PI3K: Phosphatidylinositol 3-kinase
AKT: Protein kinase B
EMT: Epithelial–mesenchymal transition
TME: Tumor microenvironment
MAPK/ERK: Mitogen-activated protein kinase/extracellular signal-regulated kinase
JAK/STAT: Janus kinase/signal transducer and activator of transcription
OR: Odds ratio
HR: Hazard ratio
IC50: Half maximal inhibitory concentration
p-AKT: Phosphorylated AKT
p-ERK: Phosphorylated ERK
mTOR: Mechanistic target of rapamycin
FFPE: Formalin-fixed, paraffin-embedded
